# Supplementary material for: Analysis of flavonol regulator evolution in the Brassicaceae reveals MYB12, MYB111 and MYB21 duplications and MYB11 and MYB24 gene loss
Source: BMC Genomics. 2022 Aug 19;23:604. doi: 10.1186/s12864-022-08819-8 (PMC9392221; doi:10.1186/s12864-022-08819-8)
Supplement: Supplementary file 3 — Additional file 3. [file 12864_2022_8819_MOESM3_ESM.pdf]

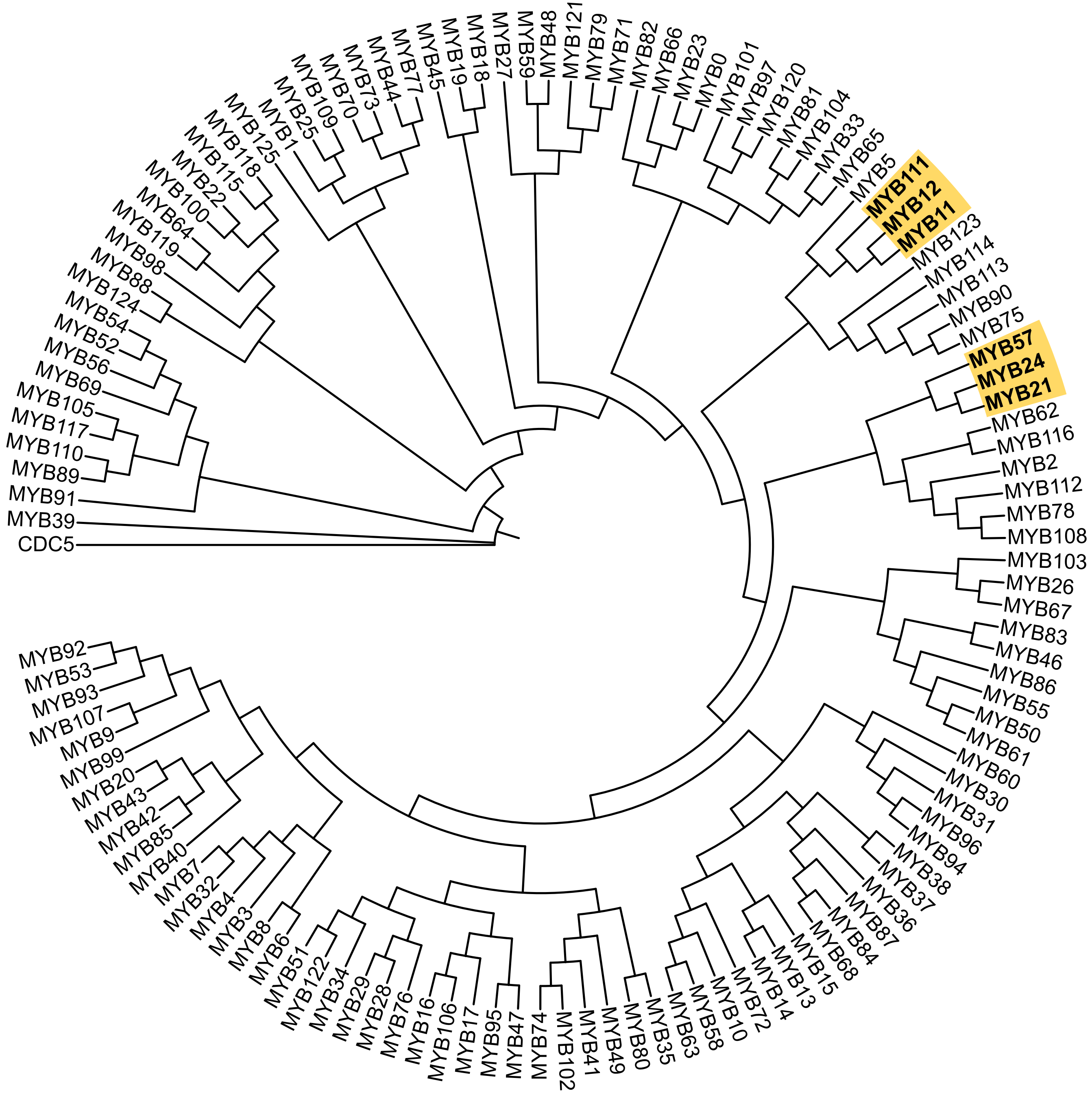

Additional file 3: Schematic overview of the R2R3-MYB phylogeny of *Arabidopsis thaliana*. The subgroup 7 MYBs (MYB11, MYB12, MYB111) and subgroup 19 MYBs (MYB21, MYB24, MYB57) are shown in bold highlighted in yellow. The full amino acid sequences were aligned with ClustalW. MEGA version 11.0.11 was used to perform neighbor-joining tree analysis with 1,000 bootstraps.
