# Supplementary material for: Analysis of flavonol regulator evolution in the Brassicaceae reveals MYB12, MYB111 and MYB21 duplications and MYB11 and MYB24 gene loss
Source: BMC Genomics. 2022 Aug 19;23:604. doi: 10.1186/s12864-022-08819-8 (PMC9392221; doi:10.1186/s12864-022-08819-8)
Supplement: Supplementary file 4 — Additional file 4. [file 12864_2022_8819_MOESM4_ESM.pdf]

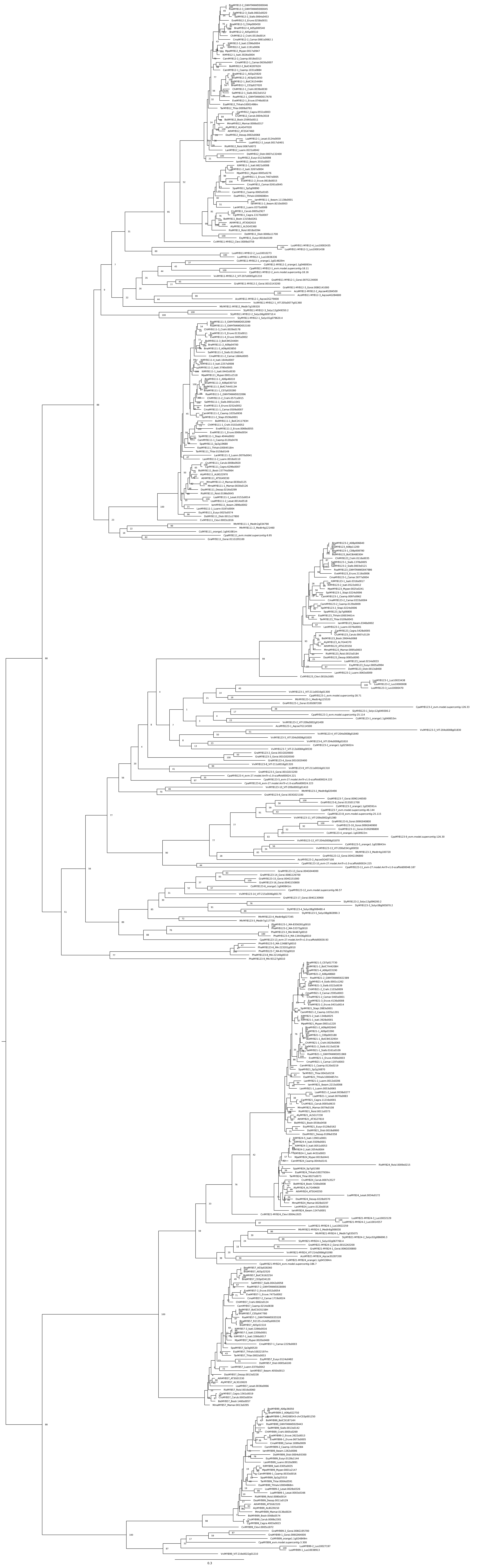

Additional file 4: Phylogenetic tree of SG5, SG7, SG19 and MYB99 members. Bootstrap values are represented as percentages.
