# Supplementary material for: Analysis of flavonol regulator evolution in the Brassicaceae reveals MYB12, MYB111 and MYB21 duplications and MYB11 and MYB24 gene loss
Source: BMC Genomics. 2022 Aug 19;23:604. doi: 10.1186/s12864-022-08819-8 (PMC9392221; doi:10.1186/s12864-022-08819-8)
Supplement: Supplementary file 8 — Additional file 8. [file 12864_2022_8819_MOESM8_ESM.pdf]

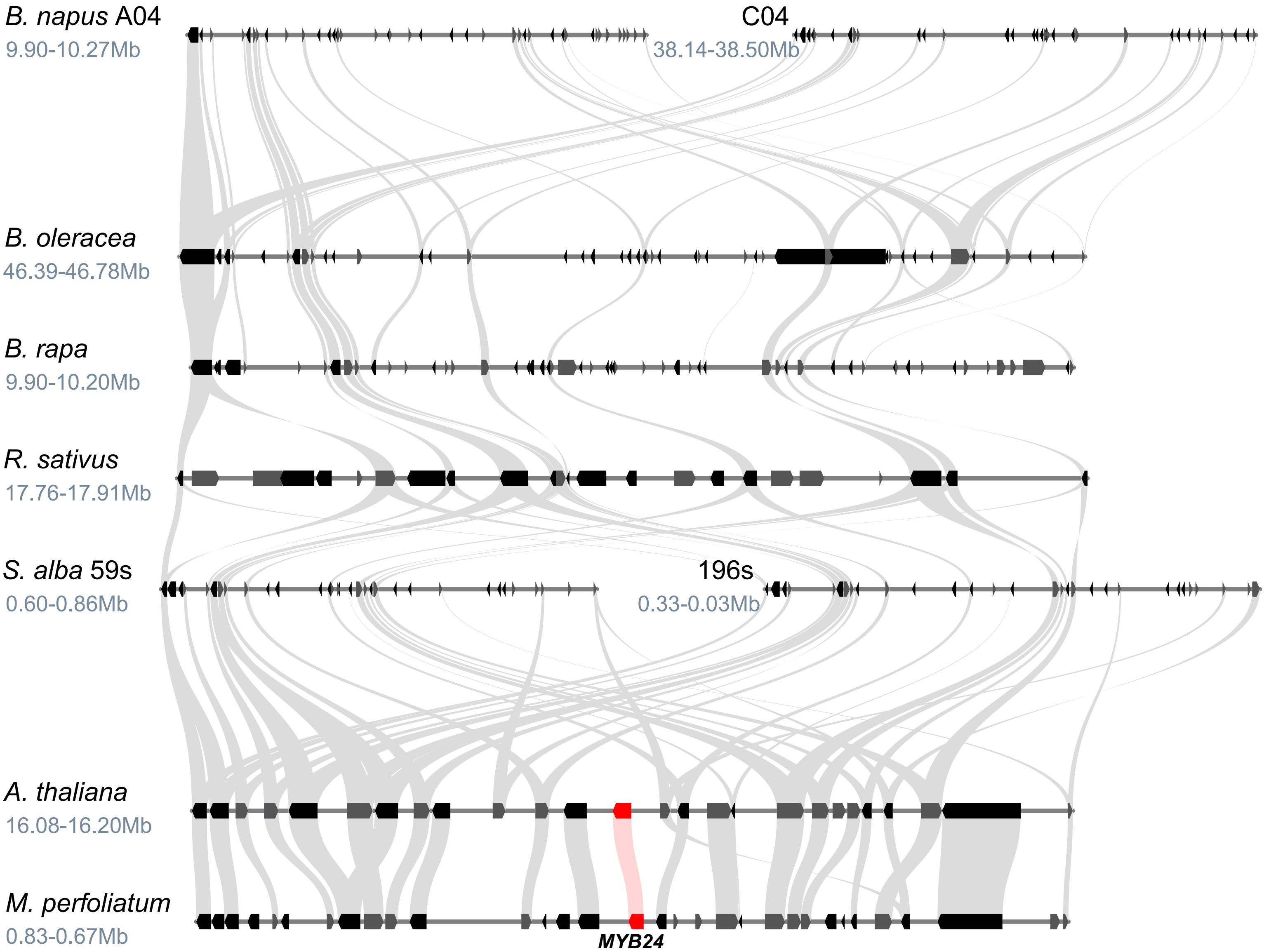

Additional file 8: Synteny analysis of the *MYB24* locus including the second *S. alba* high local synteny locus.
